# Behavioral intentions and perceived stress under isolated environment

**DOI:** 10.1002/brb3.3347

**Published:** 2023-12-31

**Authors:** Ying Lin, Lili Wu, Hui Ouyang, Jingye Zhan, Jing Wang, Weizhi Liu, Yanpu Jia

**Affiliations:** ^1^ China Executive Leadership Academy Pudong Shanghai China; ^2^ Laboratory for Post‐traumatic Stress Disorder, Faculty of Psychology and Mental Health Naval Medical University Shanghai China; ^3^ The Emotion & Cognition Laboratory, Faculty of Psychology and Mental Health Naval Medical University Shanghai China; ^4^ Key Laboratory of Molecular Neurobiology of the Ministry of Education Naval Medical University Shanghai China

**Keywords:** behavioral intentions, compulsory measures, perceived stress, quarantine duration

## Abstract

**Background:**

Isolation is a special environment that will affect the mental health and behavior of individuals. The current study was to explore the relationship between behavior intention (BI) and perceived stress in isolated environment during Shanghai Omicron pandemic.

**Methods:**

A cross‐sectional study was conducted between April 8 and 14, 2022. Three self‐reported questionnaires were used to evaluate quarantine duration, stress perception, and BI. A total of 1042 participants in Shanghai under quarantine at home were included by random sampling. Logistic regression and one‐way variance analysis were used to determine the risk factors related to BI.

**Results:**

The finding implicated negative BI was more reported by the population of males, with lower educational background, with jobs, and youngers. A negative association existed between perceived stress and BI (*B* = −1.004, *p* = .003, OR = 0.367, 95% CI = .191–.703). The proportion of positive BI decreased with quarantined duration, whereas the negative BI seemed vibrate upward then downward.

**Conclusion:**

There existed a significant effect of quarantined days on perceived stress with different BIs. High perceived stress was a risk factor of positive BI. This preliminary study has significance to understand the effect of compulsory measures on BI and for policies makers to take a psychosocial perspective to consider the effective pandemic intervention strategies.

## Graphical abstract

This preliminary study was to see the feasibility of behavioral intentions (BI) as an indirect evaluation on psychosocial endurance, by exploring the relationship between BI and perceived stress, of individuals isolated. There existed a significant effect of quarantined days on perceived stress with different BIs. High perceived stress was a risk factor of positive BI. Understanding the effect of compulsory measures on BI may inform future effective pandemic intervention strategies.

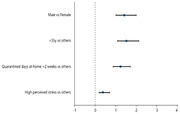

## 1 LIMITATIONS

First, this cross‐sectional survey was conducted from April 8 to 14, 2022, with an average quarantine duration of 20 days, when the measures of quarantine at home with the whole city lockdown were implemented in Shanghai. We estimated the lockdown would be lifted soon, and thought it was the right time to ask the question: “what are the three things you most want to do once the home quarantine is over?” In fact, the lockdown lasted for about another 50 days and was over until June 1st. It will be better to conduct the survey on the behavior intention (BI) of public in mid‐May, with the longer duration of compulsory measures, as more negative psychological responses like social emotions and social opinions were observed. Nevertheless, the results of the current study did reflect the true BI and perceived stress of the participants who had been quarantined at home for an average of 20 days. Second, this preliminary study was to speculate on the relationship between stress perception and BI in pandemic, by BI, which was the self‐estimation about the probability of performing a certain behavior once the lockdown was lifted, but not judge directly by the behaviors happened when really unlocked. The latter may be more representative.

However, due to the isolation during the Omicron pandemic in Shanghai, we did not know the exact time of unlocking, so we cannot assess behaviors directly. It was necessary to explore the indirect evaluation as an antecedent for the policies maker to take a comprehensive consideration on the pandemic intervention strategy.

## 2 INTRODUCTION

Isolation is a special environment that will affect the mental health and behavior of individuals. Many studies highlighted the great negative impact of isolated environment on individuals’ mental health, especially for the vulnerable groups, by the threat on emotional states (Hou et al., 2021; Yang et al., 2021). Studies have explored the effects of prison environments on the mental health of prisoners, showing that prolonged isolation can lead to poor mental health and intense feelings of anger, frustration, and anxiety (Nurse et al., 2003). As a completely closed environment of extreme pressure, long‐term space missions not only brought pressure to astronauts but also affected individual emotions, cognition, and sleep (Arone et al., 2021). Studies on the daily office environment also showed that the more people in each enclosed office space, the more adverse effects on health (Herbig et al., 2016). Although these studies investigated different subjects and backgrounds, they all consistently showed that isolation environment would have different degrees of impact on individual mental health. It is of great significance to explore the emotional state and behavioral performance in the isolation environment to improve the quality of life of people in this background.

Especially after the outbreak of the new epidemic, in order to reduce the impact of the virus on production and life, various countries introduced strict personnel control measures. Previous outbreaks of SARS, MERS have demonstrated the negative impact of isolation on mental health, with isolation leading to increased psychiatric symptoms, especially stress reactions, such as anxiety, depression, and distress. In recent years, measures like lockdown, quarantine, and social distancing taken by many countries in response to COVID‐19 pandemic have all led to people living in isolated environments for long periods. These isolation measures were associated with varying levels of restriction on movement, social interactions, and economic activities that were introduced in society, mostly by government, to reduce or completely eliminate disease transmission (Eyawo et al., 2021). Some studies showed that such isolated measures reduced COVID‐19 mortality and healthcare demand in some countries, such as the United States, United Kingdom, South Korea, Iran, France, and China, by modeling of the impact of these non‐pharmaceutical interventions (Hsiang et al., 2020). And isolations, though effective, also imposed significant social and economic cost on individuals and societies (Eyawo et al., 2021). Based on the study of pandemic responses in the United States and the United Kingdom, it was point out the existence of a numbing effect of lockdown groupthink that united in crowds, with the observations on lockdowns affecting many millions of people with poverty, food insecurity, loneliness, unemployment, school closures, and interrupted healthcare (Joffe, 2021). Therefore, how to maintain positive BIs in such isolated environment is not only important for individual health but also for national stability.

Behavioral intention is considered to be the immediate antecedent of behavior and refers to the self‐estimation that a person makes about the probability of performing a certain behavior (Padilla‐Bautista & Galindo‐Aldana, 2022). It is a core concept in the theory of planned behavior model, which has provided predictive answers to health‐disease problems, such as behavioral intention to stay under lockdown (Padilla‐Bautista & Galindo‐Aldana, 2022), social distancing intention during COVID‐19 pandemic (Hagger et al., 2020), or vaccination intention (Schmid et al., 2017). By telling whether the public's BI are more in basic needs hierarchy or advanced, it can be indicated the potential strength and resilience of psychosocial endurance of the compulsory measures in pandemic. Specifically, if the reporting BI falls in more basic needs hierarchy, it will be coded as negative response representing the challenge of psychosocial endurance. Otherwise, the BI corresponding with high‐level needs (such as belongingness and love needs, esteem needs, and self‐actualization need) will be coded as positive response representing some extent of psychological adaption and acceptance. A comprehensive evaluation of the psychosocial endurance toward the compulsory measures is necessary, considering both the proportion of negative BI (bottom line) and positive BI (social capacity) in the population.

Stress perception is an important factor that may influence BI during the COVID‐19 pandemic. The greater the pressure, people tend to show more negative behavior or idea. For example, studies showed that nurses experienced a higher level of distress and burnout and used more maladaptive coping strategies (Lou et al., 2022). Occupational stress had a direct and positive relationship with turnover intention (Jiang et al., 2022). In addition, during epidemic, perceived severity and fear of COVID‐19 were positively associated with vaccine acceptance, whereas higher level of risk exposures (work/study place exposure) and negative attitude toward general vaccination were associated with low vaccine acceptance (Qiao et al., 2022). Therefore, it was believed that people's perceived pressure will have an impact on BI in isolated environment led by COVID‐19.

The purpose of this preliminary study was to explore the relationship between BI and perceived stress in isolated environment during Shanghai Omicron pandemic. To the best of our knowledge, there were no studies have explored the relationship between perceived stress and behavioral intention under the COVID‐19. But given that previous studies have revealed that the risk perceptions of COVID‐19 negatively affected people's acceptance of vaccination (Caserotti et al., 2021; Commodari et al., 2020; Qiao et al., 2022), this article hypothesized high perceived stress was a risk factor of positive BI and quarantined days had an impact on this relationship. The results of this study may provide a useful reference for policy makers and to take a psychosocial perspective to consider the effective pandemic intervention strategies.

## 3 METHOD

### 3.1 Participants

Random sampling was employed from the residents of Shanghai where seriously hit by Omicron pandemic in China. The survey started from April 8 to 14, 2022 when the city has been put on lockdown and people have been quarantined due to the pandemic spreading. It was conducted an online survey by using the Questionnaire Star platform (https://www.wjx.cn) which was a publicly available platform in China. The survey was mainly distributed to the society through daily broadcasting on WeChat groups and psychological service institutions. On opening the link of the survey, there was an announcement to clarify the purpose of the study and then all the items for the study for those who volunteered to respond. This design was to make the participants clear about what private information needed to be provided before they decided to join in.

We received 1320 participants through survey. The inclusion criteria involved (1) willing to accept the investigation; (2) located in Shanghai; (3) normal ability of speech, comprehension and expression; (4) >18 years; (5) without a history of mental illness. Two hundred twenty‐eight were deleted because of not meeting the inclusion criterion and 50 were deleted for too much missing values (38 removed) or spending more than 30 min or less than 2 min to response the survey (12 removed). Finally, 1042 pieces of data were used in the statistical analysis. As the *F*‐test of the highest three groups was involved in this article, the power package in R language was used to analyze the sample size required for the study, and there was a preset medium effect size *f* = .25, 1 − *β* = .8 in the statistical test, and *α* = .05 in the significance level. The results showed that at least 53 subjects were required in each group. Therefore, the sample size in this paper meets the minimum sample size requirements. This study was approved by the ethics committees of [edited out for blind review].

### 3.2 Measures

#### 3.2.1 Demographic characteristics

Demographic information, including age, gender, occupation, and education, was collected.

#### 3.2.2 Duration of quarantine

It was measured by quarantined days at home, which the participants would calculate and fill in according to the individual situation by the date answering the questionnaire.

#### 3.2.3 Stress perception

During the epidemic, people faced a variety of stressors, including fears of infection and inadequate supplies (Brooks et al., 2020), but few previous studies have made that distinction. Therefore, to get a clear picture of the relationship between a particular stress and behavioral intention during the Omicron pandemic in Shanghai, stress perception was assessed by 10 items involving the general stress perception and 9 specific perceptions on the stressors, including living provisions, risk of COVID‐19 infection, family dysfunction, children learning online, medical or medicine demand, economic income, negative information, cramped space, and working at home, which were integrated from the previous studies (Brooks et al., 2020; Wilken et al., 2017) and according to the actual situation in Shanghai during quarantine. Just as one study involving three questions to investigate respondents’ risk perception toward three diseases from 0 (not at all likely) to 100 (extremely likely) (Caserotti et al., 2021), the stress perception questionnaire was also a self‐reported nine‐point Likert scale designed to investigate people's perceived stress. All perceptions were ranged from 1 (no stress at all) to 9 (an enormous stress). The average score of 10 items was calculated to represent the state of stress perception, meaning people with higher score had higher perceived stress.

#### 3.2.4 Behavioral intentions

To obtain everyone's BI when the lockdown was lifted, the participants need to answer a question: “what are the three things you most want to do once the home quarantine is over?” We sorted out keywords and assigned them according to the following principles. Higher scores indicating greater positive cognition and attitudes about caring for patients with infectious diseases were shown in Cui's study (Cui et al., 2022). Similarly, based on this classification of BI, authoritative person coded BI was positive or not according to its emotional color in the present study. Specifically, three researchers coded the answers for positive and negative valence, based on the key words in the answers. For example, if answers representing some basic needs unmet or deprived under lockdown situation were coded as negative, such as the demand of seeking medical treatment and dispensing, resting, and sleeping. In case of disagreement in coding results, we invited an authoritative person to conduct coding and synthesized the opinions to make the final assignment. After three answers were coded, participants with two or three negative intentions were considered negative BI, one negative intention as neutral BI, and nonnegative intentions as positive BI.

## 4 STATISTICAL ANALYSIS

Statistical analysis was performed using IBM SPSS version 21.0. A two‐tailed test was used, and *p* < .05 was considered statistically significant. Descriptive and frequency statistics (mean, [SD], and percentages) were used to describe baseline demographic information. First, descriptive statistics were calculated for the demographic variables based on different groups. Chi‐square test was applied to analyzed differences of BI by demographic information and perceived stress. Then, a logistic regression was used to determine the risk factors related to positive BI. Lastly, one‐way variance analysis (ANOVA) was used to detect the effect of quarantined days at home on perceived stress of population with different BI.

## 5 TRANSPARENCY AND OPENNESS

The data that support the findings of this study are available from the corresponding authors upon reasonable request.

## 6 RESULTS

### 6.1 Demographic information

Participants’ demographic characteristics were reported respectively in Table 1. There were 621 males and 421 females included. As for quarantined days at home, the highest response rate was 2–3 weeks (36.9%, *n* = 384), whereas the lowest was <2 weeks (26.6%, *n* = 276). As for BI, there were 13.9% (*n* = 145) participants reporting negative BI and 76.5% (*n* = 797) reporting positive BI. Significant association in age (*χ* ^2^ = 10.341, *p* = .035) and quarantined days at home (*χ* ^2^ = 11.046, *p* = .026) was found in different BI groups. Among them, people of the age of <35 showed both higher percentage of negative and positive BI than other ages. In the population of negative BI, more people with <35 years reported negative BI (60.69%, *n* = 88), which was showed in Table S1.

**TABLE 1:** Demographic information of participants.

		*n* (%)	Behavior intention(BI)				
			Negative BI	Neutral BI	Positive BI	*χ* ^2^	*p*
**Total**		1042(100)	145(13.9%)	100(9.6%)	797(76.5%)		
**Gender**							
	Male	621(59.6)	93(15%)	57(9.2%)	471(75.8%)	1.605	.448
	Female	421(40.4)	52(12.4%)	43(10.2%)	326(77.4%)		
**Age**							
	<35	619(59.4)	88(14.2%)	45(7.3%)	486(78.5%)	10.341	.035*
	35–55	388(37.2)	53(13.7)	49(12.6%)	286(73.7%)		
	>55	35(3.4)	4(11.4%)	6(17.1%)	25(71.5%)		
**Education**							
	High school or less	142(13.6)	23(16.2%)	13(9.2%)	106(74.6%)	2.537	.864
	Junior college	294(28.2%)	46(15.6%)	29(9.9%)	219(74.5%)		
	Bachelor degree	323(31)	42(13.0%)	31(9.6%)	250(77.4%)		
	Master degree or above	283(27.2)	34(12.0%)	27(9.5%)	222(78.5%)		
**Personnel category**							
	People with jobs	898(86.2%)	128(14.3%)	87(9.7%)	683(76.0%)	4.950	.292
	Students	63(6%)	4(6.3%)	4(6.3%)	55(87.4%)		
	Retired or unemployed personnel	81(7.8%)	13(16.1%)	9(11.1%)	59(72.8%)		
**Quarantined days at home**							
	<2 week	277(26.6%)	36(13.0%)	22(7.9%)	219(79.1%)	11.046	.026*
	2–3 weeks	384(36.9%)	63(16.4%)	28(7.3%)	293(76.3%)		
	>3 weeks	381(36.6%)	46(12.1%)	50(13.1%)	285(74.8%)		

^*^ *p* < .05.

### 6.2 The effect of perceived stress on BI

**FIGURE 1:**
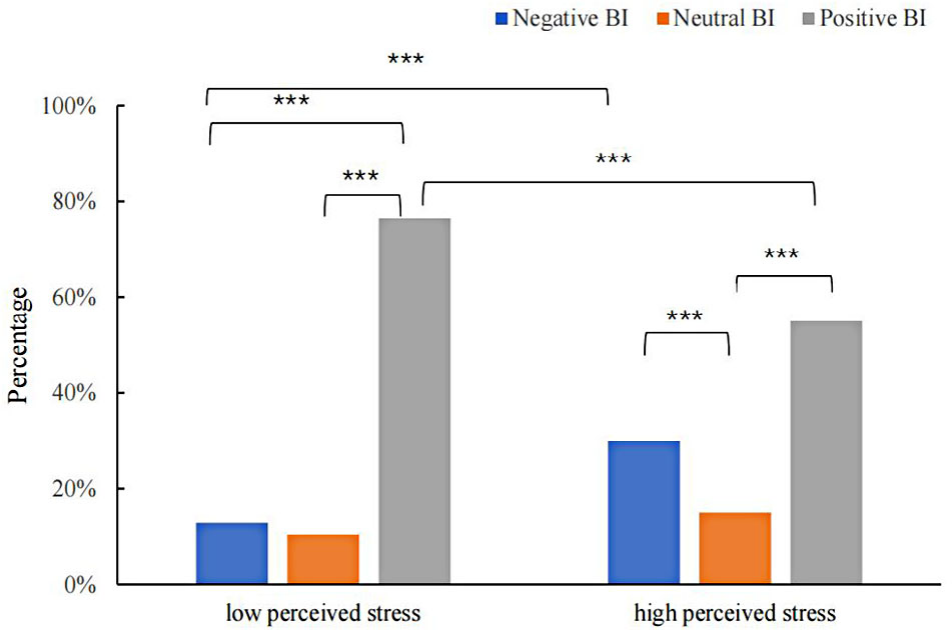
The effect of perceived stress on behavior intention.

According to the average score of perceived stress, it was divided into three groups for further analysis (Figure 1). The average score of perceived stress <4 coded as low and >7 as high. The results of Chi‐square test showed that the significant association in perceived stress (*χ* ^2^ = 10.837, *p* = .004). Specifically, low perceived stress reports more positive BI than high perceived group. Table S2 further shows that, among people with negative BI, age was significantly related with different perceived stressors. It was indicated the perceived stress of older people mainly comes from the overall stress, living provision, risk of COVID‐19 infection, family dysfunction, medical or medicine demand, economic income, negative information, cramped space, and working at home.

### 6.3 Risk factors related to positive BI

**FIGURE 2:**
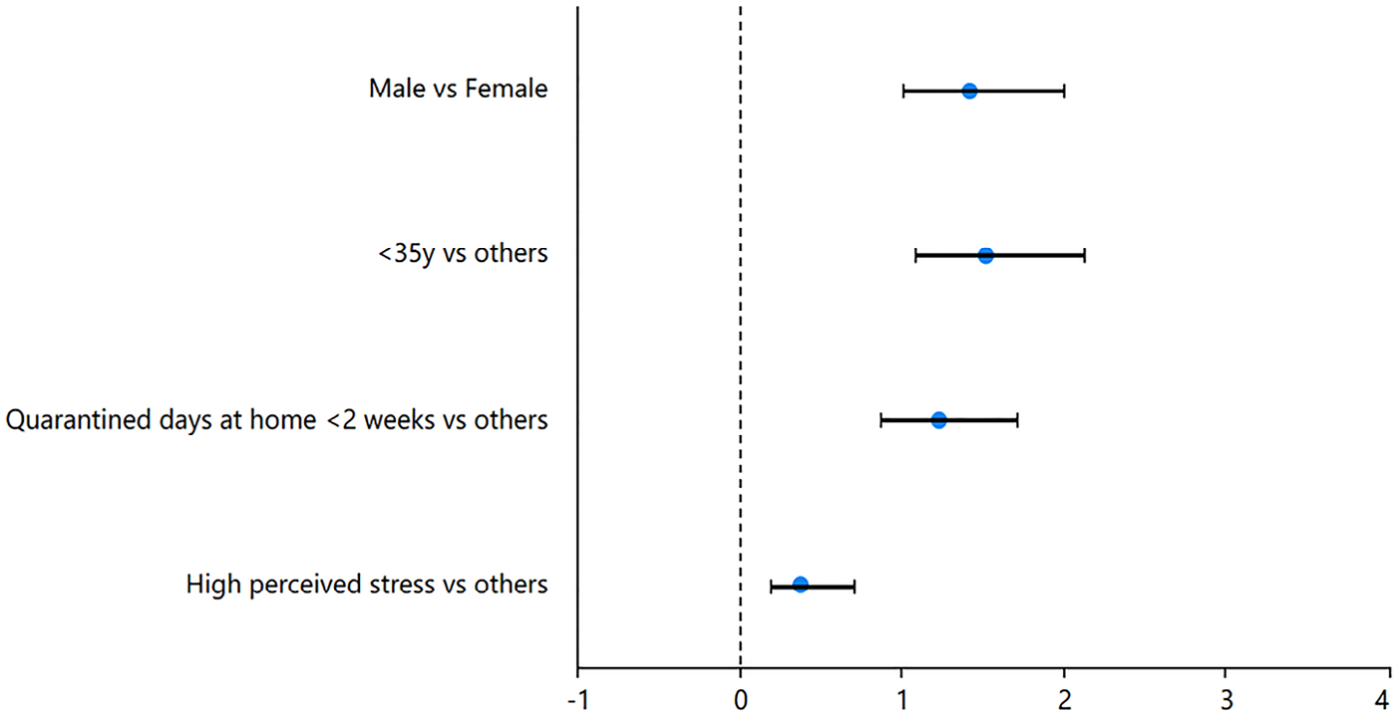
Odd ratios (95% CI) of people with positive behavior intention (BI).

To discover related factors with the development of BI, we divided it into two variables according to whether there was positive BI or not. That is, we fused previously negative and neutral BI as the no‐positive BI group. According to the results of Chi‐square test, we selected the significant characteristics and incorporated them into the logistic regression model. The results of the logistic regression analysis were listed in Table 2 and Figure 2. And the results suggested that low age was a protective factor of positive BI (*B* = .412, *p* = .018, OR = 1.510, 95% CI = 1.074–2.123, and VIF = 1.356), whereas Table S1 shows there were more people with the age of <35 (*χ* ^2^ = 73.669, *p* < .001) in negative BI group. These seemingly contradictory results suggested that the younger (for example: <35 years) had a two‐level differentiation in BI, either positive or negative, and less neutral. High perceived stress was a risk factor of positive BI (*B* = −1.004, *p* = .003, OR = 0.367, 95% CI = 0.191–0.703, VIF = 1.022), meaning participants with higher perceived stress would exhibit less positive BI. Although the effect of quarantined days at home was not significant to positive BI in the regression, when we analyzed the proportion of negative BI on different sampling date, it was found that the resonance existed between the sampling dates and negative BI (Figure S1).

**TABLE 2:** Logistic regression analysis for the risk of positive behavior intention (BI).

Predictor of positive BI	*B*	*SE*	*z*	Wald *χ* ^2^	*p*	OR	95% CI	VIF
Male vs. female	.346	.177	1.951	3.808	.051	1.413	.998–1.999	1.354
<35 years vs. others	.412	.174	2.369	5.615	.018	1.510	1.074–2.123	1.356
Quarantined days at home <2 weeks vs. others	.199	.172	1.157	1.338	.247	1.221	.871–1.711	1.012
High perceived stress vs. others	−1.004	.333	−3.017	9.104	.003	0.367	.191–0.703	1.022
Intercept	.445	.138	1.432	2.052	.152	1.576	.846–2.938	

### 6.4 The effect of quarantined days at home on perceived stress with different BI

**FIGURE 3:**
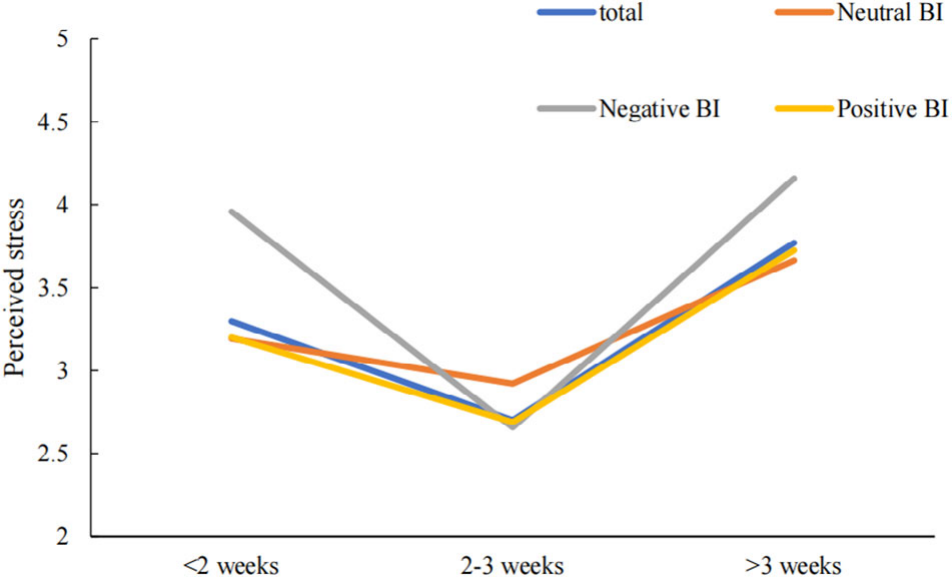
The effect of quarantined days at home on perceived stress of population with different behavior intention (BI).

The results of ANOVA showed significant effect of quarantined days at home on perceived stress of population with different BI. In general, there existed significant effects between the relationship of quarantined days at home and perceived stress (*F* = 30.846, *p* < .001, *η* ^2^ = .056), showing a downward trend and then upward trend of perceived stress over quarantined days and the stress of >3 weeks was significantly higher than <2 weeks (*p* = .004, *η* ^2^ = .012), so did the positive (*p* = .007, *η* ^2^ = .014) group. Although the negative BI group showed the same trend of changes, there was no significance between stress of >3 weeks and that of <2 weeks (*p* = .287, *η* ^2^ = .014). It had to say that perceived stress in the neutral BI group did not change significantly with the quarantined days. And from Figure 3, we can see that, at the beginning of quarantine at home, higher stress was reported on those with more negative BI (M (negative BI) = 4.003, M (neutral BI) = 3.182, M (positive BI) = 3.322, and M (total) = 3.400).

## 7 DISCUSSION

The current study aimed to explore the relationship between BI and perceived stress under Shanghai home quarantine during the outbreak of Omicron pandemic. It was showed that negative BI was more reported by the population of males, with lower educational background, with jobs, and youngers. A negative association existed between perceived stress and BI. Logistic regression revealed that high perceived stress was a risk factor of positive BI. The proportion of positive BI decreased with quarantined duration, whereas the negative BI seemed vibrate upward then downward. The observation of BIs across the phase of the isolation allows important considerations regarding psychological, duration of quarantine, and susceptible factors useful to tailor public health intervention to improve public response to future epidemics.

Several groups of significant differences were found in the proportion of negative BI. According to the demographic results with negative BI (in Table S1), it was suggested that negative BIs were more reported by the population of males, younger (aged <35), with junior college educational background, and with jobs. This result can be explained by previous researches. Such as, the finding of previous study revealed that youngers were more vulnerable to perceive stress during COVID‐19 pandemic (Varma et al., 2021). And statistically significant differences were noted in mean perceived stress between gender, younger compared to older students, and those without compared to those with previous higher education qualification (Naidoo & Pau, 2008). Because of the close connection between behavior and emotion, similar results were found for behavioral intention. The impacting factors behind the demographic results need to be discussed furtherly, whereas the relative previous studies may shed a light on it. For example, the study in Germany showed that positive pandemic appraisal, social support, and adaptive cognitive emotion regulation were positively related to mental health outcomes under COVID‐19 lockdown (Ahrens et al., 2021). And the evidences from social and behavioral sciences showed negative framing usually captures attention, especially for the people with strong emotional responses (Bavel et al., 2020). In view of the close relationship between mental health and behavior, it was necessary to carry out active intervention for susceptible people.

In addition, the results proved that a negative association existed between perceived stress and BI. What's more, it was found that high perceived stress was a risk factor of positive BI, meaning participants with higher perceived stress would exhibit less positive BI. This was consistent with previous researches that stress was influencing factors of working turnover intention (Lu et al., 2017), home‐gardening intentions (Wu et al., 2022), and defensive medical behavior (Sun et al., 2022). During the epidemic, people faced a variety of stressors, including fears of infection and inadequate supplies Just as supplementary material indicated, the stresses of older people mainly come from the overall stress, living provisions, risk of COVID‐19 infection, family dysfunction, children learning online, medical or medicine demand, economic income, negative information, cramped space, and working at home. The results of this study suggested that essential intervention and governance measures should be considered to decrease individuals’ all kinds of specific stresses, which were urgently needed.

It was worth mentioning that quarantined days at home was found to have a significant association with BI groups, showing the positive BI decreased with the quarantined duration, whereas the negative BI seemed vibrate upward then downward. In the further analysis, it showed the significant effect of quarantined days at home on perceived stress of population with different BI, with the general finding of the downward trend and then upward trend of perceived stress over quarantined days, which result was consistent with the previous study indicating that the duration of quarantine was obviously a stressor during quarantine (Brooks et al., 2020; Hou et al., 2021). Therefore, the government should consider the impact of quarantine time on people's stress and behavior in the face of other outbreaks in the future.

It was interesting to see the different effect of quarantine duration on perceived stress between different BI groups. Specifically, the positive BI group showed a downward trend and then upward trend of perceived stress over quarantined days and the stress of >3 weeks was significantly higher than <2 weeks. Although the negative BI group showed the same trend of changes, there was no significance between perceived stress of >3 weeks and that of <2 weeks, with highest stress reported at the beginning of quarantine. And the perceived stress in the neutral BI group did not change significantly with the quarantined days. It seemed tell us that the different BI group showing the different baseline levels of perceived stress at the beginning of quarantine, which may be related to the inner resources of the subject, the important impacting factors of psychosocial endurance of outside stimulus (Dajun Zhang, 1997). For example, it was highlighted that the various cognitive factors mediated the relationship between stimulus and emotion in the previous study, which found that inner resources like dispositional optimism, uncertainty tolerance, and social support could buffer the direct or indirect effects of quarantine length on depression and anxiety during quarantine (Hou et al., 2021). In this sense, in order to improve the emotional and behavioral performance of individuals, the government should adopt different isolation times according to different groups of people in the face of future epidemics.

## 8 CONCLUSION

This preliminary study provided us with the findings that a negative association existed between perceived stress and BI groups, and quarantined days at home also had a significant association with BI groups, showing the proportion of positive BI decreased with the quarantined duration, and the negative BI seemed vibrate upward then downward. The present study has significance for us to understand the effect of isolated environment on BI, to furtherly discuss the impacting factors of BI, and for policies maker to take a psychosocial perspective to consider the effective pandemic intervention strategy.

## AUTHOR CONTRIBUTIONS

Ying Lin contributed to the writing of this article and was the first author. Lili Wu, Weizhi Liu, and Yanpu Jia leaded the whole study, including putting forward this study, carrying out the study, and the statistical analysis of this article, and they were the co‐corresponding author. Hui Ouyang, Jingye Zhan, and Jing Wang contributed to collection of all data.

## CONFLICT OF INTEREST STATEMENT

The authors declared no potential conflicts of interest with respect to the research, authorship, and/or publication of this article.

### PEER REVIEW

The peer review history for this article is available at https://publons.com/publon/10.1002/brb3.3347.

## Supporting information


**Table S1** Demographic information of participants with negative BI.
**Figure S1** The percentage of negative BI over time.
**Table S2** Correlations between age and different perceived stresses in negative BI.

## Data Availability

The data that support the findings of this study are available from the corresponding authors upon reasonable request.
