# Supplementary material for: Behavioral intentions and perceived stress under isolated environment
Source: Brain Behav. 2023 Dec 31;14(1):e3347. doi: 10.1002/brb3.3347 (PMC10757889; doi:10.1002/brb3.3347)
Supplement: Supplementary file 1 — Table S1 Demographic information of participants with negative BI. Figure S1 The percentage of negative BI over time. Table S2 Correlations between age and different perceived stresses in negative BI. [file BRB3-14-e3347-s001.docx]

**Supplementary material**

In order to provide theoretical support for future interventions and policies, we further investigated the influencing factors in those with negative BI. The findings of supplementary material will support the main conclusion of current study.

**Demographic information of participants with negative BI**

We selected participants with negative BI out to visualize the differences in the proportion of negative cBI in each demographic variable. Chi-square test showed that more male (χ2 = 11.593, *p* = .001), people with the age of <35(χ2 = 73.669, *p* < .001), people with junior college educational background (χ2 = 8.517, *p*= .036), people with jobs (χ2 = 197.807, *p* < .001) and quarantined days at home 2-3 weeks (χ2 =7.710, *p* = .021) reported negative BI. But the results of logistic regression suggested that low age was a protective factor of positive BI (*B*=0.412, *p*=.018, OR=1.510, 95%CI=1.074 - 2.123). while Table S1 showed people with the age of <35(χ2 = 73.669, *p* <.001) showed more negative BI. These results suggested that young people (for example: <35y) had a two-level differentiation in BI, either positive or negative, and less neutral.

| **TABLE S1**  *Demographic information of participants with negative BI* | | | | | |
| --- | --- | --- | --- | --- | --- |
|  |  | **Negative BI (n=145)** | | **χ2** | ***p*** |
|  |  | **n** | **%** |  |  |
|  |  |  |  |  |  |
|  |  |  |  |  |  |
| **Gender** |  |  |  |  |  |
|  | male | 93 | 64.14 | 11.593 | .001** |
|  | female | 52 | 35.86 |  |  |
| **Age** |  |  |  |  |  |
|  | <35 | 88 | 60.69 | 73.669 | <.001*** |
|  | 35-55 | 53 | 36.55 |  |  |
|  | >55 | 4 | 2.76 |  |  |
| **Education** |  |  |  |  |  |
|  | high school or less | 23 | 15.86 | 8.517 | .036* |
|  | junior college | 46 | 31.72 |  |  |
|  | bachelor degree | 42 | 28.97 |  |  |
|  | master degree or above | 34 | 23.45 |  |  |
| **Personnel category** |  |  |  |  |  |
|  | people with jobs | 128 | 88.28 | 197.807 | <.001*** |
|  | students | 4 | 2.76 |  |  |
|  | retired or unemployed personnel | 13 | 8.97 |  |  |
| **Quarantined days at home** |  |  |  |  |  |
|  | <2 week | 36 | 24.83 | 7.710 | 0.021* |
|  | 2-3 weeks | 63 | 43.45 |  |  |
|  | >3 weeks | 46 | 31.72 |  |  |

Note: **p* < .05. ***p* < .01. ****p* < .001.

**The change of negative BI over different sampling days**

Although the effect of quarantined days at home was not significant in the logistic regression, when we analyzed the proportion of negative BI in different sampling days, it was found that the resonance change between sampling dates and negative BI (supplementary material Figure S1). Specifically, the proportion of negative reports was the largest at the beginning, and it eased in the middle, and then showed an upward trend with the increase of sampling dates.

**FIGURE S1**. *The percentage of negative BI over time*

**Correlations between age and different perceived stresses in negative BI**

Previous results had evidenced that a significant relationship between perceived stress and negative BI. Then, pearson correlation was used to further discuss source of stress in those with negative BI. The pearson correlation proved that, among people with negative cognition, age was significantly related with different stresses. It was indicated the stresses of older people mainly comes from the overall stress, living provisions, risk of COVID-19 infection, family dysfunction, children learning online, medical or medicine demand, economic income, negative information, cramped space, and working at home.

| **TABLE S2**  *Correlations between age and different perceived stresses in negative BI* | | | | | | |  |  |  |  |  |  |
| --- | --- | --- | --- | --- | --- | --- | --- | --- | --- | --- | --- | --- |
|  |  | 1 | 2 | 3 | 4 | 5 | 6 | 7 | 8 | 9 | 10 | 11 |
| 1.Age |  | 1.000 |  |  |  |  |  |  |  |  |  |  |
| 2.Stress from overall |  | .489** | 1.000 |  |  |  |  |  |  |  |  |  |
| 3.Stress from living provisions |  | .520** | .807** | 1.000 |  |  |  |  |  |  |  |  |
| 4.Stress from risk of COVID-19 infection |  | .554** | .691** | .712** | 1.000 |  |  |  |  |  |  |  |
| 5.Stress from family disfunction |  | .190* | .615** | .574** | .534** | 1.000 |  |  |  |  |  |  |
| 6.Stress from children learning |  | .161 | .353* | .345** | .328** | .454** | 1.000 |  |  |  |  |  |
| 7.Stress from medical or medicine demand |  | .390** | .572** | .587** | .552** | .474** | .453** | 1.000 |  |  |  |  |
| 8.Stress from economic income |  | .169* | .499** | .526** | .425** | .568** | .451** | .492** | 1.000 |  |  |  |
| 9.Stress from negative information |  | .349** | .623** | .606** | .575** | .577** | .541** | .505** | .621** | 1.000 |  |  |
| 10.Stress from cramped space |  | .322** | .726** | .650** | .652** | .627** | .374** | .542** | .490** | .728** | 1.000 |  |
| 11.Stress from working at home |  | .170* | .609** | .558** | .463** | .500** | .441** | .452** | .463** | .551** | .630** | 1.000 |

Note: **p* < .05. ***p* < .01.
